# Case for diagnosis. Unilateral nodule on the nipple: erosive adenomatosis of the nipple^☆^

**DOI:** 10.1016/j.abd.2022.09.014

**Published:** 2023-07-08

**Authors:** Raúl Gerardo Mendez-Flores, Karen Uriarte-Ruiz, María Elisa Vega-Memije, Daniela Ruiz-Gomez, Sonia Toussaint-Caire

**Affiliations:** a Department of Dermatopathology, General Hospital “Dr. Manuel Gea González”, Mexico City, Mexico; b Department of Dermatology, General Hospital “Dr. Manuel Gea González”, Mexico City, Mexico

Dear Editor,

A 44-year-old Hispanic woman came to our dermatology clinic with an asymptomatic exophytic lesion on her right nipple that had been present for two years. There was no family history of breast cancer. The lesion started as a small erythematous plaque that gradually grew and developed a focal erosion. On dermatological examination, a 1.0 × 1.0 cm hard erythematous ill-defined lesion with a central erosion was noticed. Dermoscopy showed pink-white clouds and red structureless areas (Fig. 1). There was no lymphadenopathy or nipple retraction.

**Figure 1:**
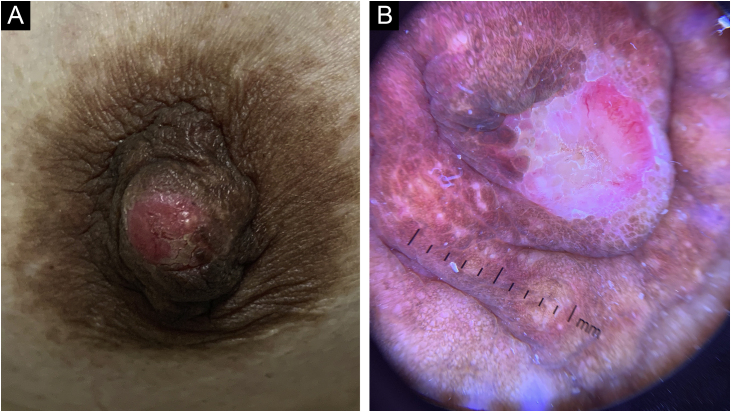
(A) A firm nodule with a central erosion on the right nipple. (B) Dermoscopy showed pink-white clouds and red structureless areas

An incisional biopsy of the right nipple was performed. Histopathology revealed a well-circumscribed dermal tumor with adenomatous and papillary configuration. The tumor consisted of multiple ductal structures lined by a double layer of columnar eosinophilic cells, some of which showed secretion by cell decapitation. A basal layer of myoepithelial cells was present. A ductal opening communicated with the surface epithelium at one end. No cellular atypia or pleomorphism was noticed (Fig. 2).

**Figure 2:**
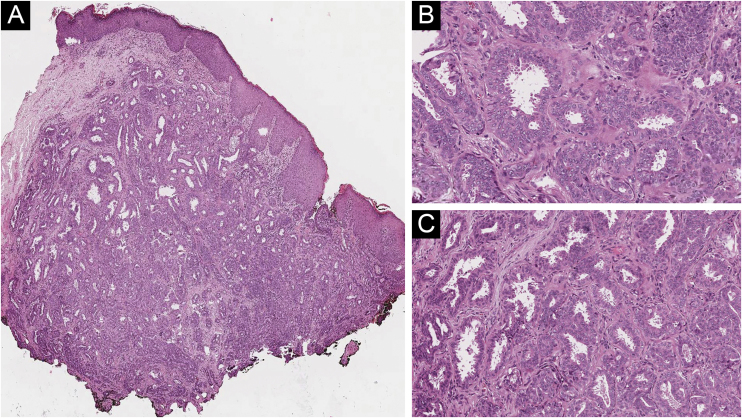
(A) Histology showing a well-circumscribed, non-encapsulated dermal glandular proliferation (Hematoxylin & eosin, ×100). (B‒C) Ductal structures lined with cuboidal epithelial cells, which present apocrine secretory projections on its luminal border (Hematoxylin & eosin, ×400)

## What is your diagnosis?

a) Paget's disease of the breast;
b) Contact dermatitis;
c) Ductal carcinoma;
d) Erosive adenomatosis of the nipple.

## Discussion

Erosive Adenomatosis of the Nipple (EAN), also known as nipple adenoma, papillary adenoma of the nipple, or florid papillomatosis, is an uncommon benign epithelial tumor that originates from the lactotrophic ducts of the nipple-areola complex. It was first reported as a benign intraductal papilloma in 1951 by Haagensen et al.^1^, ^2^

Like our patient, it affects middle-aged women, with an average age of 43 to 45 years which is uncommon in men.^2^

Clinically it presents as an erythematous unilateral mass with a partial or complete erosion with serous or serosanguineous discharge. In advanced stages, the nipple becomes enlarged, thickened, and indurated and may present as a big exophytic mass.^3^

Mammary Paget disease may also present with a nipple tumor with erosion and serosanguineous discharge, and it is often associated with ductal carcinoma in situ.^4^ Thus, the most important differential diagnoses include mammary Paget's disease and breast ductal carcinoma, however other inflammatory (contact dermatitis eczema) and infectious diseases may mimic EAN.

The histological findings are the most valuable evidence in differentiating EAN from these inflammatory and malignant mammary tumors. Histopathology reveals a glandular, well-circumscribed, non-encapsulated proliferation coated by a characteristic double layer of cells composed of an external layer of cubic or flattened myoepithelial cells and an internal layer of cuboidal or cylindrical epithelial cells, which can present apocrine secretory projections on its luminal border.^1^ The absence of cytologic atypia is an important feature.

Surgical excision with nipple resection is the therapy of choice. The Mohs micrographic surgery and nipple splitting enucleation procedure can entirely remove the tumor while preserving the appearance and functionality of this vital location.^4^ Other authors have reported favorable treatment outcomes with cryosurgery and photodynamic therapy.^5^, ^6^

Identification of this lesion is critical because of clinical and therapeutic implications, unnecessary mastectomy or extensive surgeries may be carried out if misdiagnosed. Our patient was treated with complete excision of the tumor with no recurrence in a 1-year follow-up.

## Financial support

None declared.

## Authors' contributions

Raúl Gerardo Mendez Flores: Writing of the manuscript or critical review of important intellectual content; effective participation in the research guidance; final approval of the final version of the manuscript.

Karen Uriarte Ruiz: Critical review of the literature; writing of the manuscript or critical review of important intellectual content.

María Elisa Vega Memije: Effective participation in the research guidance; intellectual participation in the propaedeutic and/or therapeutic conduct of the studied cases.

Daniela Ruiz Gomez: Critical review of the literature; writing of the manuscript or critical review of important intellectual content.

Sonia Toussaint Caire: Critical review of the literature; writing of the manuscript or critical review of important intellectual content.

## Conflicts of interest

None declared.
